# Potassium Improves Drought Stress Tolerance in Plants by Affecting Root Morphology, Root Exudates, and Microbial Diversity

**DOI:** 10.3390/metabo11030131

**Published:** 2021-02-24

**Authors:** Qiwen Xu, Hao Fu, Bo Zhu, Hafiz Athar Hussain, Kangping Zhang, Xiaoqing Tian, Meichun Duan, Xiaoyu Xie, Longchang Wang

**Affiliations:** 1 Key Laboratory of Eco-Environments in Three Gorges Reservoir Region, College of Agronomy and Biotechnology, Southwest University, Chongqing 400715, China; xqiwen@yeah.net (Q.X.); ezhubo@sina.com (B.Z.); eeeezai@sina.com (K.Z.); jemesouviensxu@sohu.com (X.T.); duanmc@swu.edu.cn (M.D.); xiexy8009@sina.com (X.X.); 2 Key Laboratory of Horticulture Science for Southern Mountains Regions of Ministry of Education, College of Horticulture and Landscape Architecture, Southwest University, Chongqing 400715, China; fh1994@126.com; 3 Institute of Environment and Sustainable Development in Agriculture, Chinese Academy of Agricultural Sciences, Beijing 100081, China

**Keywords:** potassium, drought stress, root morphology, organic acids, microbes

## Abstract

Potassium (K) reduces the deleterious effects of drought stress on plants. However, this mitigation has been studied mainly in the aboveground plant pathways, while the effect of K on root-soil interactions in the underground part is still underexplored. Here, we conducted the experiments to investigate how K enhances plant resistance and tolerance to drought by controlling rhizosphere processes. Three culture methods (sand, water, and soil) evaluated two rapeseed cultivars’ root morphology, root exudates, soil nutrients, and microbial community structure under different K supply levels and water conditions to construct a defensive network of the underground part. We found that K supply increased the root length and density and the organic acids secretion. The organic acids were significantly associated with the available potassium decomposition, in order of formic acid > malonic acid > lactic acid > oxalic acid > citric acid. However, the mitigation had the hormesis effect, as the appropriate range of K facilitated the morphological characteristic and physiological function of the root system with increases of supply levels, while the excessive input of K could hinder the plant growth. The positive effect of K-fertilizer on soil pH, available phosphorus and available potassium content, and microbial diversity index was more significant under the water stress. The rhizosphere nutrients and pH further promoted the microbial community development by the structural equation modeling, while the non-rhizosphere nutrients had an indirect negative effect on microbes. In short, K application could alleviate drought stress on the growth and development of plants by regulating the morphology and secretion of roots and soil ecosystems.

## 1. Introduction

Climate change induces abiotic stressors which are the major threat to plant growth and productivity under the natural environment. Plants respond the changing climate to enhance persistence under new environmental conditions through ecological strategies such as phenotypic plasticity or evolving adaptations as rapidly as possible [1]. Climate change has led to frequent weather extremes and unstable water supply resulted in the trend of normalization of droughts [2]. The United Nations pointed out in the World Water Resources Integrated Assessment Report that water resources will become a significant limiting factor for global economic and social development, with the potential to trigger conflicts and contradictions among countries [3]. It is estimated that losses caused by drought stress account for 40–60% of the total crop yield loss globally [4], indicating that water deficit is the main factor of crop failure among all abiotic stresses. Currently, agriculture accounts for 70% of total freshwater consumption, most of which is used for crop production [5], prompting the urgent need to improve water use efficiency and drought resistance of agricultural production crops.

Fertilization strategies promote plant growth, anatomy, morphology, plant metabolism and improve nutrient reserves to cope with adverse environmental conditions [6]. Potassium (K) is an essential mineral nutrient for plants, which holds the key for many physiological processes in different crop species, like photosynthesis, protein synthesis, stomatal movement, and osmoregulation [7,8,9]. Promising evidence showed that the significant relationship between soil K and plant water. Plants can absorb K from the soil to improve their water use efficiency [7], maintaining the tolerance and resistance at a high level to withstand drought stress. The positive effect of K is to alter the CO_2_ input process by regulating the stomatal function, which alleviates the photo-assimilation limited by water deficit [8,10], and K also controls carbohydrate metabolizing enzymes to enhances the accumulation and translocation of sucrose [11], all of those processes are closely related to plant growth and productivity. However, most studies on K have focused on the effects of the aboveground part of plants while how the underground part of plants and soil ecosystems are driven by K application and plant growth is largely unknown.

As the first organ in plants to perceive drought stress is the root system morphology, which is not only determined by their genes but also influenced by surrounding environmental changes [12]. When plants are exposed to water-limited conditions, the root biomass and extension are inhibited [13], and lateral roots developing and root angle forming are structurally adjusted to the distribution of soil moisture [14]. In addition to such physical adaptations, roots also release certain organic compounds into the soil to improve the rhizosphere environment, which is considered as a “stress-reducing” mechanism during the long-term evolution of plants [15,16] and medium between plants and soil [17,18]. Meanwhile, organic acids in root exudates can increase the available mineral nutrients in the soil to support the nutrient uptake and transport for plants [19]. In addition to roots, the close link between microbial communities and plant growth establish in soil ecosystems [1,20,21]. As microbes with semipermeable membranes are in close contact with soil water, the small cells water potential is prone to balance with the surrounding water potential [22]. When microorganisms suffered drought stress, the cells reduce their internal water potential by accumulating the solutes to prevent dehydration and death [23]. On the other hand, soil microbial communities also predominate in plant adaptation to drought stress. For example, there is higher soil nitrogen availability in wet-adapted microbial communities than those in dry-adapted microbial communities [1]. Sufficient availability of nutrients helps to maintain proper growth during the adverse period [11], whereas plant nutrient absorption is affected by self-adaptations of microbial community composition [24]. Nevertheless, it is poorly understood whether the root system’s response and soil microbe to drought will change with K intervention and contribution.

Here, we combined three culture methods, sand, hydroponics, and soil culture, to investigate physiological and ecological strategies of plants and more comprehensive mechanisms of drought alleviated by K from the perspective of the underground part. Instead of employing one culture method, we used sand culture to emphasize the process of root system morphology, followed by hydroponics to obtain intact roots to collect root exudates and, thus, reduce disturbances of bleeding sap from broken roots and soil particles attached to roots surface. The aims were to investigate the development of root systems and organic acids under water deficit with K intervention and see the differences in activation of soil nutrients by root exudates. Simultaneously, we designed root bags in pots of soil culture to distinguish between rhizosphere and non-rhizosphere soil, which could emphasize the nutrient acquisition strategies of the roots system under adversity. Furthermore, in this way, we expect to find whether K would build tolerance or resistance with plants by affecting the microbial community, establishing a root-nutrient-microbial interaction.

## 2. Result

### 2.1. Experiment I

#### 2.1.1. Root Morphological Characters under Different K Supply Levels

**Figure 1:**
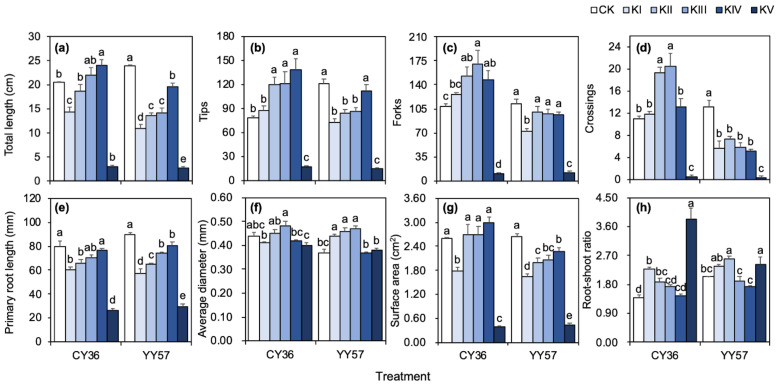
Effects of K supply level on the root system architecture: (**a**) total length, (**b**) the number of tips, (**c**) the number of forks, (**d**) the number of crossing, (**e**) primary root length, (**f**) average diameter, (**g**) surface area, (**h**) root-shoot ratio of two rapeseed cultivars. KI, KII, KIII, KIV, and KV indicate K supply level (K_2_SO_4_) 0, 0.1, 1, 10, and 100 mM under drought stress with 15% PEG, respectively, and CK indicate the control (without 15% PEG) 0.1 mM K_2_SO_4_. Means and standard errors (*n* = 4). Different small letters mean significant differences in Duncan multiple range tests among different treatments at 5% level (*p* < 0.05).

The effects of water stress on two cultivars under different: drought stress were significantly decreased the primary root length of CY36 and the total length, the number of tips and crossings, primary root length, and surface area of YY57 when compared CK with treatment-KII (Figure 1). Instead, the number of tips, forks, and crossings of the CY36 (Figure 1) and the root average diameter of YY57 (Figure 1f) was significantly increased by drought. Whereas the water stress was relieved by inputting K, which K supply significantly increased eight root morphological indices in Figure 1 of CY36 and those indexes excluded the crossing and average diameter of YY57 compared the absence (KI) with presence (KII–KIV) of K. Those positive effects of K enhanced with the increasing does of K supply and the treatment-KIV recorded the highest level of total root length, tips, primary root length and surface area in both CY36 and YY57 (Figure 1). Interestingly, treatment-KV significantly inhibited the root morphological development in two cultivars. In contrast, the root-shoot ratio decreased with the increase of K concentration and treatment-KV recorded the maximum (Figure 1).

#### 2.1.2. Changes in the Quantity and Composition of Organic Acids with K Supply

**Figure 2:**
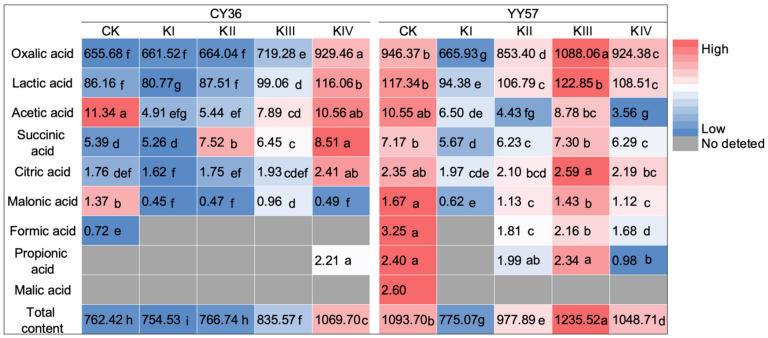
Effects of K supply level on the organic acids in root exudates of two rapeseed cultivars. KI, KII, KIII, and KIV indicate K supply level (K_2_SO_4_) 0, 0.1, 1 and 10 mM under drought stress with 15% PEG, respectively, and CK indicate the control (without 15% PEG) 0.1 mM K_2_SO_4_. The numbers indicate the average content of organic acids (*n* = 4). Different small letters mean significant differences in Duncan multiple range tests among different treatments at 5% level (*p* < 0.05).

In this experiment, nine organic acids were detected by HPLC in rapeseed root exudates: oxalic acid, lactic acid, citric acid, succinic acid, malonic acid, acetic acid, propionic acid, formic acid, and malic acid. Apparently, there were more kinds of organic acids in YY57 root exudates than those in CY36 (Figure 2). Drought inhibited the exudation of malic acid in YY57 and formic acid in CY36 when compared CK with treatment-KII, and decreased the quantity of organic acids in YY57 (Figure 2). Compared to treatment-KI, organic acids content significantly increased with increasing amount of K (excluded malonic acid of CY36), but the trend was recorded differently in two cultivars: YY57 was in the order of KIII > CK > KIV > KII > KI (*p <* 0.05) and CY36 was in the order of KIV > KIII > KII > CK > KI, which the maximum difference was up to 59.41% and 41.77%, respectively (Figure 2). For the composition of organic acids, the absence of K inhibited the secretion of formic acid and propionic acid in YY57, while the higher supply level of K stimulated the exudation of propionic acid in CY36 (Figure 2).

#### 2.1.3. Soil Available K Activated by Organic Acids

**Figure 3:**
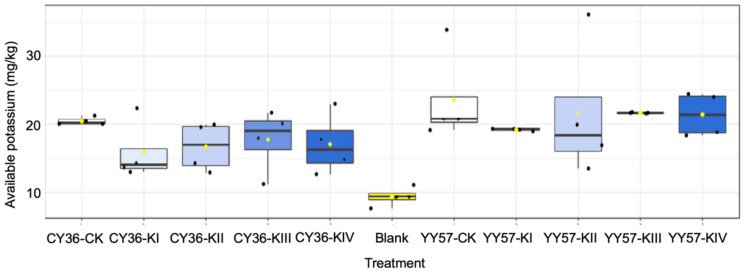
Effects of organic acids on AK activating in two rapeseed cultivars. Here the value of AK is the difference before and after adding the organic acid extract, KI, KII, KIII, and KIV indicate K supply level (K_2_SO_4_) 0, 0.1, 1 and 10 mM under drought stress with 15% PEG, respectively, and CK indicate the control (without 15% PEG) 0.1 mM K_2_SO_4_. The blank is water treatment to compare with the extracts. The black dots represent the maximum, upper quartile, lower quartile, and the minimum, and yellow dots represent the median. Means and standard errors (*n* = 4).

**Figure 4:**
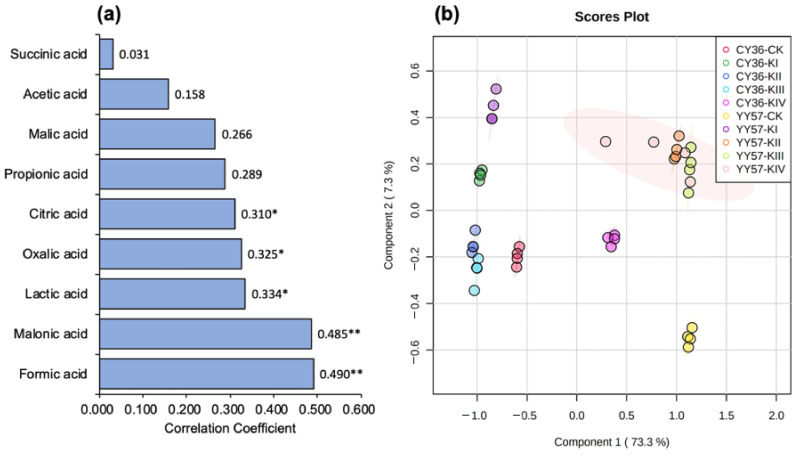
(**a**) The pattern search of nine organic acids in root exudates to activate AK. The numbers indicate the correlation coefficients between organic acids and the activation of AK. ** *p <* 0.01; * *p <* 0.05. (**b**) The partial least squares discriminant analysis of restoration evaluation in two rapeseed cultivars. KI, KII, KIII, and KIV indicate K supply level (K_2_SO_4_) 0, 0.1, 1, and 10 mM under drought stress with 15% PEG, respectively, and CK indicate the control (without 15% PEG) 0.1 mM K_2_SO_4_.

Compared to the blank, root exudates extract significantly increased the activation content of AK by 87.3% and 129.2% of CY36 and YY57 (*p* < 0.05) on average, respectively (Figure 3). Available K (AK) content in CK was higher than this in drought treatments (KI-KIV), by 17.7% of CY36 and 11.1% of YY57 (Figure 3). Applying K increased mobilization of soil AK, by 8.2% of CY36 and 12.4% of YY57 on average when the present (KII-KIV) compared to the absence (KI) of K (Figure 3). The pattern search further indicated that nine organic acids positively correlated with the activation of soil AK (Figure 4a). Formic acid and malonic acid were the best predictors of AK mobilizing (*p <* 0.01), followed by lactic acid, oxalic acid, and citric acid (*p <* 0.05) (Figure 4a).

#### 2.1.4. Partial Least Squares Discriminant Analysis

To determine the mitigative effect of K on drought stress, partial least squares discriminant analysis (PLS-DA) was conducted separately on each sample according to root morphological characteristics and organic acids. A clear separation of samples into four quadrants was achieved by principal components, which explained 73.3% by PC1 and 7.3% by PC2 of the total variation (Figure 4b). The KII, KIII, and KIV treatments in YY57 were separated in the first quadrant (Figure 4b). The KI-treatment in YY57 and CY36 were similar, which were scattered throughout the second quadrant. The KII and KIII treatments of CY36 were separated in the third quadrant with those CK (Figure 4b). Moreover, PLS-DA divided the KIV-treatment of CY36 with the CK of YY57 in the last quadrant (Figure 4b).

### 2.2. Experiment II

#### 2.2.1. Physicochemical Properties of Rhizosphere and Non-Rhizosphere Soil

**Figure 5:**
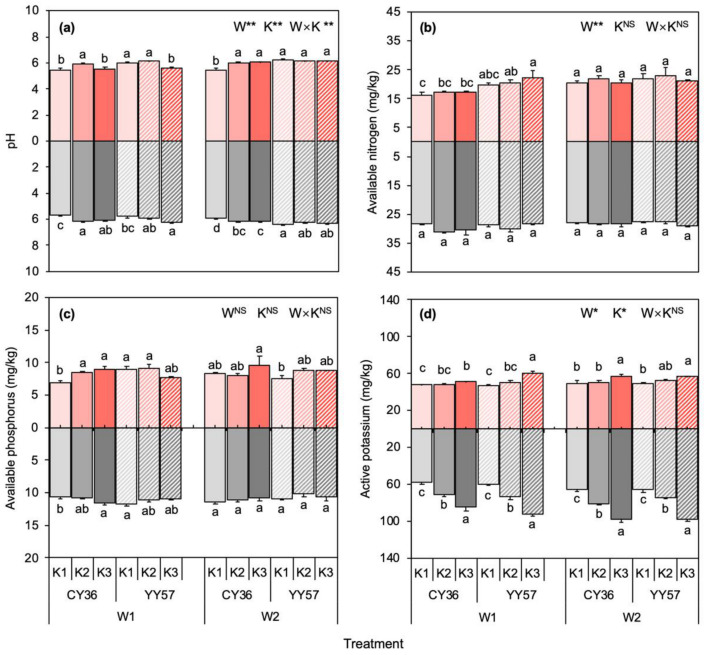
Effects of K supply level on the rhizosphere (values above *x*-axis) and non-rhizosphere (values below the *x*-axis) (**a**) pH, (**b**) available nitrogen, (**c**) available phosphorus, and (**d**) available potassium content of two rapeseed cultivars under different soil moisture content. W1 and W2 indicate 40% and 75% water-holding capacity, respectively, and K1, K2, and K3 indicate K (K_2_O) applications rates 0, 80, and 160 mg·kg^−1^, respectively. Means and standard errors (*n* = 4). Different small letters mean significant differences in Duncan multiple range tests among different treatments at 5% level (*p <* 0.05). Two-way analysis of variance (ANOVA) was performed to evaluate the effects of soil moisture content (W), K-fertilizer (K), and their interactions (W×K). NS means non-significant. * and ** indicate significant differences at *p <* 0.05 and *p <* 0.01 probability levels, respectively.

From the results obtained in Figure 5, it seems that the soil pH was intermediately acidic and pH levels of non-rhizosphere soil (5.65–6.37) were higher than those in rhizosphere soil (5.46–6.22). Soil moisture content, K-fertilizer, and their interactions showed highly significant differences (*p <* 0.01) on pH values (Figure 5a). In CY36, treatment-K2 recorded the highest pH numerical values under the water-limited condition in the rhizosphere and non-rhizosphere soil, which amount to a 0.41 and 0.52 unit significant increase, respectively, compared to treatment-K1 (Figure 5a). Similar results were observed under the water-unlimited condition that pH levels of rhizosphere and non-rhizosphere soil increased a 0.57 and 0.24 unit, respectively (*p <* 0.05) (Figure 5a). However, YY57 did not show the trends, and the trends of pH levels in the rhizosphere differ from those in non-rhizosphere soil (Figure 5a). Under the water-limited condition, treatment-K3 recorded the highest pH numerical values (6.20) in the non-rhizosphere while it also recorded the lowest level (5.62) in rhizosphere soil (Figure 5a). Mostly, no difference under water-unlimited conditions was witnessed between K1, K2, and K3 in YY57.

Soil available nitrogen (AN), available phosphorus (AP), AK levels in non-rhizosphere were also higher than rhizosphere soil, by 42.8%, 30.7%, and 47.9% on average (Figure 5). Soil moisture content significantly affected soil AN content in both rhizospheres (*p <* 0.05) and non-rhizosphere (*p <* 0.01) soil, while no significant impact of K-fertilizer on AN was demonstrated (Figure 5b). Despite no significant differences in soil AP content of YY57, applying K-fertilizer significantly increased the content of AP in CY36 under the water-limited condition, which amounts to a 29.9% and a 9.4% increase in the rhizosphere and non-rhizosphere soil, respectively, compared K3 with K1 treatments (Figure 5c). Additionally, there were significant (*p <* 0.01) impacts of soil moisture content and K-fertilizer, which resulted in increased values of soil AK (Figure 5d). This positive effect of K on soil AK content was higher in the non-rhizosphere, namely, significant differences were showed among K1, K2, and K3 in the non-rhizosphere while they were only witnessed between K1 and K3 in rhizosphere soil (Figure 5d).

#### 2.2.2. Microbial Diversity Indexes under Water-Potassium Combination

**Figure 6:**
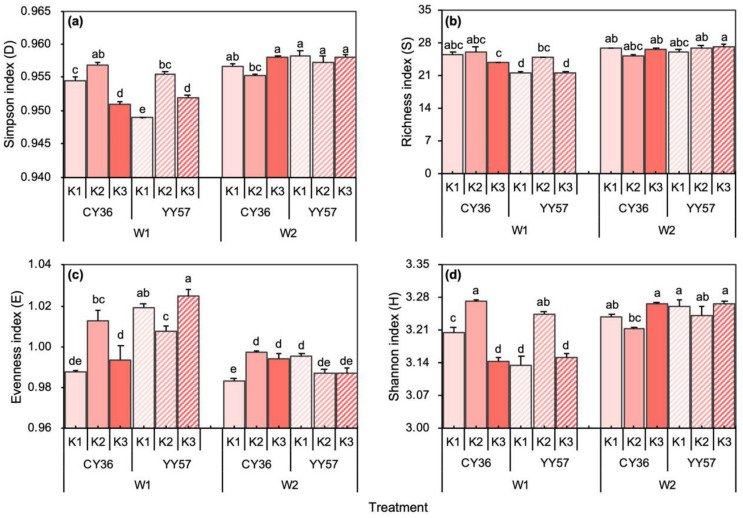
Effects of K supply level on the rhizosphere microbial diversity index under different soil moisture content: (**a**) Simpson index (*D*), (**b**) richness index (*S*), (**c**) evenness index (*E*), (**d**) Shannon index (*H*) of two rapeseed cultivars. W1 and W2 indicate 40% and 75% water-holding capacity, respectively, and K1, K2, and K3 indicate K (K_2_O) applications rates 0, 80 and 160 mg·kg^−1^, respectively. Means and standard errors (*n* = 4). Different small letters mean significant differences in Duncan multiple range tests among different treatments at 5% level (*p <* 0.05).

Analysis of variance indicated that soil moisture content, K-fertilizer, and their interaction significantly affected Simpson index (*D*), richness index (*S*), Shannon index (*H*), while the evenness index (*E*) was only significantly affected by soil moisture content (Figure 6). After 14 days of drought stress, the levels of *D*, *S*, and *H* decreased significantly by 0.27%, 4.4%, 1.03% in CY36 and 0.59%, 17.5%, 2.50% in YY57 on average compared to water-unlimited condition, but the level of *E* increased significantly by 0.67% and 2.76% of CY36 and YY57, respectively (Figure 6). Under the water-limited condition, the effect of treatment-K3 varied with different cultivars (Figure 6). The addition of K to soil in CY36 would be arranged in an ascending order of values with *D*, *S*, *E*, and *H*: K2 > K1 > K3 (*p <* 0.05) (Figure 6). This amounts to a 2.45%, a 1.2%, a 2.50%, and a 2.60% increase in the level of *D*, *S*, *E*, and *H* compared treatment-K2 with K1 (Figure 6). Treatment-K2 also recorded the highest numerical values of those in YY57, but the trend was different from CY36, which was an ascending order of *D*, *S*, and *H*: K2 > K3 > K1, increasing 6.83% (*D*), 15.4% (*S*), 3.46% (*H*) under treatment-K2 when compared to treatment-K1 (Figure 6).

#### 2.2.3. Relationship in the Soil Ecosystem

**Figure 7:**
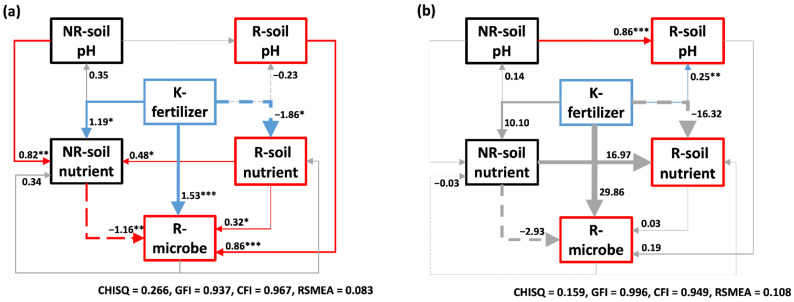
Structural equation modeling (SEM) about the effect of K supply level on soil properties of the rhizosphere (R) and non-rhizosphere (NR) and rhizosphere microbe and their interaction and relationships under (**a**) water-limited conditions and (**b**) water-unlimited condition. SEM are colored according to classification (blue for K-effect, red for R-region, and black for NR-region, while gray lines represent non-significant coefficients (*p* > 0.05). Full lines represent positive relationships, while dotted lines represent negative relationships, which the width of lines indicates the strength of the relationship. *** *p <* 0.001; ** *p <* 0.01; * *p <* 0.05.

Structural equation modeling (SEM) brought forward a solution to visualize the direct and indirect impacts of K-fertilizer and the interactions between soil properties and microbe under the different soil moisture content (Figure 7). There were more significant effects under the water-limited condition (Figure 7). A positive effect of K-fertilizer on NR-nutrient and R-microbe while a negative effect on R-nutrient was observed with drought stress (Figure 7). NR-pH and R-nutrient revealed a significant positive correlation with NR- nutrient. R-microbe under the water-limited condition was influenced by many factors, which are directly positive impacts of R pH and nutrient and a negative impact of NR-nutrient (Figure 7). However, there was only a positive significant influence of K-fertilizer on R-pH under the water-unlimited condition and we also observed that NR-pH showed a more considerable impact on R-pH than K-fertilizer (Figure 7).

## 3. Discussion

As predicted, K application was beneficial for drought-stressed plants. In our experiments, we found that K elongated the root length to obtain water from even deeper and wider soil layers and simultaneously increased the root density and surface area to expand their contact surface with the surrounding, thus helping plants efficiently uptake available water and in this way mitigating drought. Additionally, K also made the root-shoot ratio towards 1, which could balance the ratio and function of whole plants to further improve the efficiency of resource utilization [25]. Interestingly, this mitigation varies with the increase of K supply level, closely matching the concept of hormesis effect that mainly described ions of unknown physiological function [26], which assumes that the effect of an element on plant depends on its concentration [27]. K is an inorganic solute that play imperative role for osmotic potential in roots [28]: too high a supply level of it can damage the turgor-pressure-driven translocation of solute and break the water balance in plant organs [29]. The stressed root system developed healthily with increasing K concentration, and KIV was the gradient that could achieve the best recovery of rapeseed across all treatments. However, a further increase of the supply level reaching KV shifted the effect from beneficial to negative, severely limiting the root biochemical and physiological functions. In other words, the appropriate range of K facilitated the root development and metabolism, whereas the excessive input of K might trigger an imbalance of ionic homeostasis in the organism and then producing toxic effects. Different cultivars adopt distinct drought response strategies to allow itself to absorb as much water as possible and withstand the adversity: the drought-sensitive cultivar (CY36) increased the root density while the drought-tolerant cultivar (YY57) thickened the diameter of the root system (Figure 1). Concomitantly, the K alleviating effect was stronger in the drought-sensitive cultivar. The results of PLS-DA further verified that KII and KIII supply level could help the CY36 stressed root system develop comparable to its control, and CY36 even restored to YY57 control state under KIV level, while the alleviating state was similar between KII, KIII, and KIV in YY57. Those also demonstrated that plant exposed to water deficit require more internal K [30].

Significant genotypic differences also observed in the composition of organic acids. Across all treatment, malic acid, propionic acid, and formic acid widespread in the drought-tolerant cultivar (YY57) compared to the drought-sensitive cultivar (CY36), especially malic acid was only detected in the control of YY57 (Figure 2). Song et al. demonstrated that a physiological adaptation might exist in drought-tolerant cultivars to enhance nutrient solubility in the rhizosphere and mitigate the toxic effects of water stress [16]. Malic acid has been reported as a reducing matter of organic acids in root exudate, which can transform high valence into low valence metal ions to raise the efficiency of nutrients in soil [19]. Malic acid, propionic acid, and formic acid might be reasons why drought-resistant cultivars could utilize the surrounding resources more efficiently, which was also proved by the lack of those three acids in the treatment without K supply in YY57 (Figure 2). To tolerate adversity, roots could regulate matters in the soil environment by conditioning actively or passively the composition and quantity of organic acids. We found that formic acid and malic acid was inhibited in CY36 and YY57 under drought stress, respectively, which is the result of plants limited growth and development reduce the energy costs of secreting organic acids and no longer need a rich nutrients environment. Similarly, this is why a significant decline in organic acid content was observed under the water stress in two cultivars compared with those controls.

In addition to the root system architecture, K also affect the quantity and composition of organic acids in order to tackle water scarcity. K, a primary cellular osmoticum, plays a vital role in neutralizing the negative charges [7]. On the one hand, K mediated the controlled release of organic acids thought anion channels in roots as factors affecting membrane integrity may affect organic acids exudation [17]. In the 1990s, researchers identified that anion channels mediated root-controlled release of organic acids and no association between their exudation and levels within roots [31,32]. Large cytoplasmic K^+^ diffusion potential and protons create positively-charged gradients through the extrusion of ATPase, thereby stimulating the release of carboxylate anions [33], which is the reason why propionic acid and formic acid secreted extra under K supply of CY36 and YY57, respectively. On the other hand, the balance between anions and cations in the rhizosphere environment is one of the main factors of organic acids. The root process of uptaking cations (especially K^+^) is accompanied by the need of negative charges to maintain constantly the ionic equilibrium in soil environment that is usually provided by organic acids, such as malic acid, malonic acid and citric acid [34]. Interestingly, the highest content of organic acids was recorded at KIV supply level in both cultivars, which was same with the maximum points of root tip number, total root length, primary root length, and surface area. This confirmed that organic acids are mainly secreted from the tips of primary and lateral roots for active translocation and an indirect effect on organic acids from changes in root morphology due to nutrient application is in a more dominant state [35]. The regulation of root growth and branching in nutrient-rich patches areas may be consistent with root exudates increased to affect nutrient dynamics and microbial communities [11], therefore improving metabolic activities and defenses virtually [18].

Clearly, the pattern search results showed that the significant effect of organic acids on AK activation was in the order of formic acid, malonic acid, lactic acid, oxalic acid, and citric acid (Figure 4a), which confirmed that the release of non-exchangeable K could be accelerated by root exudates [9]. There were two ways of activation from organic acid on AK in soil: acidic hydrolysis and complexing dissolution. On the one hand, H^+^ dissociated in organic acids could not only promote the dissolution of insoluble minerals through acidic hydrolysis but also replaced the K from the crystal lattice to release K^+^ since the size of H3O^+^ formed by H^+^ was similar to that of K^+^ [36]. On the other hand, low-molecular-weight organic acid with carboxyl (–OH) and hydroxyl (–COOH) groups in the ortho-position tended to form metal-organic complexes with metal ions in the mineral structure [37,38], which accelerated the decomposition of soil minerals. Formic and malonic acids with acid sites in medium strength, are prone to hydrolysis as they have a weak electric field force on the H^+^ ionized. Lactic acid with –OH and –COOH, and citric acid with –COOH, are prone to complexing effects, and oxalic acid, a medium-strength acid with –COOH, is endowed with both effects [38]. This suggests that the acid strength of organic acids influences mobilization of AK in the soil as much as their complexing effect, but the conclusion was based on the drought condition as water to soil ratio was a key factor affecting the influences of organic acids [39].

In Experiment II, K-fertilized rapeseed showed a better nutritional and healthy soil environment with neutral pH, and higher content of AK and AP, which could support plants to maintain the fundamental function of metabolic processes under a water-limited condition [11]. Given that roots induced the soil environment changes, it was improbable that soil properties in the rhizosphere were similar to the non-rhizosphere. Organic acid with acidic pH secreted from roots decreases the average level of pH in the adjacent soil, which caused pH in the rhizosphere is lower than this in the non-rhizosphere (Figure 5a). Likewise, this trend was also observed in available nutrients, confirming that resources surrounding roots are consumed by plant growth, enrich microbes, and active soil animals [40]. The analysis of SEM demonstrated that microbe under drought stress were positively affected by three factors: K fertilizer with the most significant (*p <* 0.001), then pH of R-soil (*p <* 0.001) and, finally, nutrients of R-soil (*p <* 0.05) (Figure 7a). This result is in accord with the previous studies [41,42,43] that favorable pH and abundantly available nutrients provided an excellent environment for microbial development and reproduction.

In contrast to what we expected, we did not see a progressive increase in the microbial index with K inputs. K2 level recorded the highest value of Simpson index, Shannon index, and richness index in two cultivars, while K3 no longer improved those indexes and sometimes significant decreases were observed in CY36. According to the SEM, the unexpected result might mean that high K application could promote the plant and root growth better, which in turn intensified competition between roots and microbes for limited R-nutrients [40,44] to hinder the microbial growth. Meanwhile, the pH of the rhizosphere under treatment-K2 was close to a neutral environment, which is more suitable for soil community development [42]. However, in SEM, there is a negative effect on R-microbe from nutrients in non-rhizosphere soil (*p <* 0.01) and a positive effect (*p <* 0.05) of the R-nutrient on the NR-nutrient (Figure 7a). This suggests that the NR-nutrient might be indirect negative for the microbial community by competing R-nutrients, mainly because nutrients are transported from nutrient-rich rhizosphere to non-activated non-rhizosphere areas through various pathways, such as water flow, soil microbial, and animal activities. Interestingly, the effect of K-fertilizer and the water-K interactions that had been signed under drought stress was no longer significant under water-unlimited condition, and there was only a significant effect of K-fertilizer on the R-pH (Figure 7). This proved that K-fertilizer was more effective under drought condition, and plants could regulate resource demand and nutrient acquisition strategies in the underground when confronted with the environmental change to recover biochemical and physiological functions after re-watering [11].

## 4. Materials and Methods

### 4.1. Experimental Setup

#### 4.1.1. Experiment I: Effects of K Supply Level on Root Morphology and Root Exudates under Drought Stress

**Figure 8:**
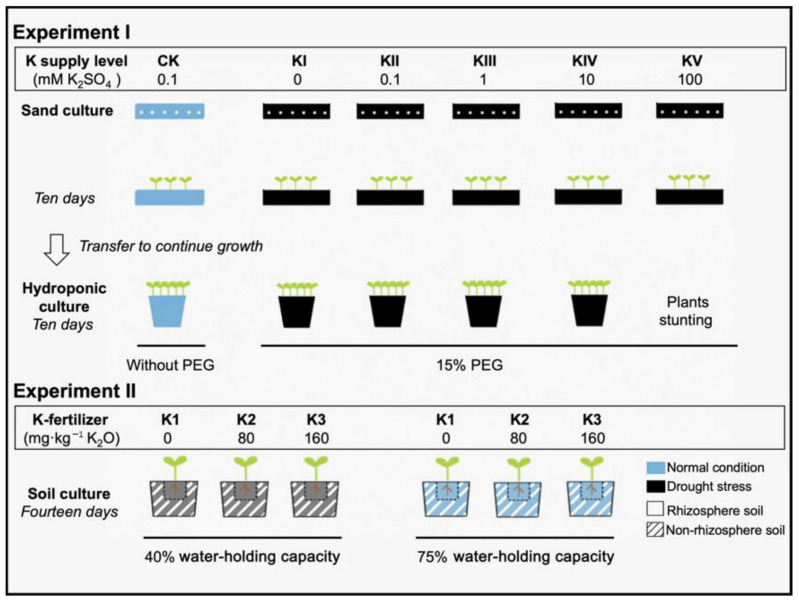
Schematic illustrating the design of Experiment I and II. Two rapeseed cultivars (CY36 and YY57) were cultured by those ways in both experiments. In the sand culture, 100 seeds were planted evenly in a germination box to establish root system architecture. In the hydroponic culture, 10-day-old rapeseed seedlings were transplanted to each pot to obtain root exudates. The treatment of Experiment I consisted of five replicated pots. In Experiment II, the dotted line record root bags that separate the rhizosphere and non-rhizosphere soil. The soil moisture treatments were started when the seedlings reached the six-leaf stage, each replicated four times.

The first experiment consisted of two phases (Figure 8), a sand culture (to establish root system architecture) and a hydroponic culture (to obtain root exudates), conducted in 2019 at the agroecology laboratory of the Southwest University, Chongqing, China (29°49′32′′ N, 106°26′02′′ E). Two rapeseed (*Brassica napus*) cultivars, CY36 (drought-sensitive) and YY57 (drought-tolerant), were selected as plant materials, which was screened from 15 rapeseed cultivars in previous work according to biomass under drought stress followed by water resupply in soil culture. The plants were supported by 1/2 strength of Hoagland nutrient solution, including macro elements, 2.5 mM Ca(NO_3_)_2_, 0.25 mM Ca(H_2_PO_4_)_2_, 1.0 mM MgSO_4_ and microelements, 20 μM FeSO_4_, 0.3 μM CuSO_4_, 0.8 μM ZnSO_4_, 5 μM MnCl, 50 μM H_3_BO_3_, 0.1 μM H_2_MoO_4_. Drought stress was simulated by adding 15% PEG6000 in the nutrient solution, and five K supply level, 0 mM, 0.1 mM, 1 mM, 10 mM, and 100 mM K_2_SO_4_, were recorded as KI, KII, KIII, KIV, KV, respectively, setting up a K application (0.1 mM) without drought stress as a control (CK). H_2_SO_4_ or Ca(OH)_2_ was used to adjust the pH to 6.0 as necessary.

In the sand culture, both cultivar seeds were disinfected by soaking in 3% NaOCl for 10 min and then thoroughly washed with deionized water. After the seeds were dried naturally, 100 seeds of both cultivars were planted evenly in plastic germination box of 15 cm length, 13 cm width, and 10 cm height with 300 g quartz sand at the bottom, approximately 2 cm thickness (Figure 8). Each germination box was supplied with 75 mL nutrient solution, which was the standard amount to cover the quartz sand at the bottom. All germination boxes with random arrange were cultured for 10 days in an illumination incubator at 25 °C, 16 h·d^−1^ light cycles, 75% humidity, and 3000 Lx light intensity. The nutrient solution level of all boxes was determined and maintained through the weighing method. After 10 days of treatment, two plants were harvested in four replicates to determine root traits.

In the hydroponic culture, five seedlings were randomly selected from each germination boxes cultured to 10 days, transferring to a black plastic pot of 120 mm diameter and 110 mm height to grow hydroponically (the treatment-KV was not transferred due to stunting). Each pot was filled with 1 L Hoagland nutrient solution and all treatments were fixed, cultivating in the same condition of illumination incubators (Figure 8). After 14 days of continued growth, the plants transfer to light-proof bottles containing 1 L of 0.5% CaCl_2_ solution for 12 h to collect root exudates behind the residues attached to the root surface were thoroughly washed with deionized water. The extracting solution was concentrated to 50 mL (20-fold concentration) using a rotary evaporator (temperature 50 °C, rpm 80–90 r·min^−1^) and stored frozen at –80 °C for the determination of organic acids and activation experiment of soil AK [45].

#### 4.1.2. Experiment II: Effects of the Water-Potassium Combination on Soil Nutrient and Microbes

The experiment was conducted in the glass greenhouse at the Southwest University, Chongqing, from September 2018 to January 2019. The soil was purple typical of southwest China, naturally dried and filtered through a 3 mm sieve. The chemical properties were as follows: 11.6 g·kg^−1^ organic matter, 0.5 g·kg^−1^ total nitrogen, 37.6 mg·kg^−1^ available nitrogen AN, 17 mg·kg^−1^ AP, 84 mg·kg^−1^ available potassium AK and 22% maximum field capacity. A split-plot experiment designed in the soil culture, in which soil moisture content (water-limited and water-unlimited) was defined as the main factor and K-fertilizer supply level (three concentration) was the assistant factor (Figure 8). The moisture level in each pot reached soil drought stress at 40% water-holding capacity (WHC) (W1) and normal condition at 75% WHC (W2). The K treatment used sulfate (K_2_SO_4_) as a fertilizer contained: 0 mg·kg^−1^ K_2_O (K1), 80 mg·kg^−1^ K_2_O (K2), 160 mg·kg^−1^ K_2_O (K3), summing to six treatments: W1K1, W1K2, W1K3, W2K1, W2K2, W2K3, each treatment included two cultivars (CY36, YY57) same with Experiment I, each replicated four times. Nitrogen and Phosphorus fertilizers were applied at 800 mg N and 400 mg P_2_O_5_ per pot, respectively, both before sowing.

A root bag made of 300 mesh nylon sieve (14 cm in length, width and height) was designed in each pot to separate the rhizosphere and non-rhizosphere soil [46]. A pot with an inner diameter of 28 cm and height of 18 cm contained 10 kg of the soil mentioned above, of which 2 kg soil were held in a root bag and 8 kg residual soil were outside (see Figures S1 and S2 in Supplementary Materials). Five rapeseed seeds were spotted per root bag on 21 September 2018 and then thinned out seedlings after one month of emergence, keeping two seedlings per pot. All treatments were maintained at a normal moisture supply during the early stages of rapeseed growth, starting soil moisture treatment of 14 days when the seedlings reached the overwintering stage (six-leaf stage). Samples were taken at the end of the treatment.

### 4.2. Measurements

#### 4.2.1. Root Morphological Traits

In Experiment I, the primary root length and shoot height were recorded. The remaining fresh sample was used for scanning root morphological parameters by an Epson perfection V700 photo scanner (Epson, Nagano, Japan), and root morphological analysis was carried by a Win RHIZO system (Regent Instrument Inc., Quebec, QC, Canada). Root-shoot ratio was calculated using the length of the primary root and shoot.

#### 4.2.2. Organic Acids in Root Exudates and Activation on Soil AK

HPLC Shimadzu LC-20AD (Shimadzu, Kyoto, Japan) was used in Experiment I to determine the secretion of organic acids in root exudates, focusing on nine organic acids: oxalic acid, tartaric acid, formic acid, malic acid, malonic acid, citric acid, succinic acid, propionic acid, and lactic acid [47]. Activation on soil AK was calculated by adding the root exudates extracting solution to 2.50 g of original air-dried soil at a ratio of 1:1.5 with deionized water as a blank control and incubated at 26 °C for 10 days in a constant temperature incubator [48].

#### 4.2.3. Soil Sampling and Physicochemical Properties

Soil samples were taken separately from rhizosphere and non-rhizosphere soil at the end of Experiment II. By this time, roots were occupied the root bag entirely, ensuring that the soil remaining after removing the top 2 cm layer of the bag was the rhizosphere sample and the soil outside the bag was the non-rhizosphere sample. All samples were quickly passed through a 2 mm sieve to remove rootlets and gravels, in which one part of the samples was stored after dried naturally to measure soil nutrients, and the other part was stored at 4 °C in a refrigerator to determine microbe.

Soil physicochemical properties were determined for both rhizosphere and non- rhizosphere. A glass electrode determines soil pH with a soil: water ratio of 1:10 (*w*/*v*). AN was obtained by the alkaline hydrolysis diffusion method. AP was detected using the molybdenum-blue method after extracted with sodium bicarbonate. AK was measured by atomic absorption spectrophotometer after extracted with ammonium acetate [49].

#### 4.2.4. Soil Microbial Community Functional Diversity

In Experiment II, soil microbial community functional diversity was determined for rhizosphere only by Biolog EcoPlate^TM^ (Biolog Inc., Hayward, CA, USA) that were incubated at 28 °C for one week [50]. Microbial data from incubations up to 120 h were selected to calculate the, Simpson dominance index (*D*), richness index (*S*), and evenness index (*E*), Shannon diversity index (*H*), calculated as follows: D = 1 − ∑ *P*i^2^, *S* = the total number of carbon sources utilized, *E* = *H*/ln *S*, *H* = −∑ *P*i (ln *P*i), where *P*i is the ratio of the relative absorbance value of i hole to the sum of those of the whole plate. *S* is the number of holes in the ECOplate with a color change when the absorbance value is less than 0.25 [51].

### 4.3. Statistical Analysis

We performed the data of both experiments were analyzed using one-way analysis of variance (ANOVA) for significant differences among treatments and Duncan’s new multiple range method for multiple comparisons using IBM SPSS 25.0 software (SPSS Inc., Chicago, IL, USA). PLS-DA was used to assess the results of drought mitigation by different concentrations of K-treatment based on the data of root morphology and organic matter content in Experiment I. Differences in the content of AK before and after addition of root exudates extracting solution was used to indicate the ability of organic acids to decompose the AK in soil. Pattern search was used to rank the contribution of nine organic acids in root exudates to activate AK. PLS-DA and pattern search were conducted by MetaboAnalyst 5.0 (https://www.metaboanalyst.ca, accessed on 23 January 2021). In Experiment II, we carried out ANCOVAs to explore soil moisture, K supply level, and their interactive effects on soil properties and microbial community in IBM SPSS 25.0 software. Structural equation modeling (SEM) was used to determine the direct and indirect contributions of K-fertilizer to rhizosphere and non- rhizosphere soil properties and microbial communities and their internal structure and causal relationships with each other [52]. The SEM fitness was proved on the index of a non-significant chi-square test (*p* > 0.05), the goodness of fit index (GFI), the comparative fit index (CFI), and the root mean square error of approximation (RMSEA) using the lavaan package carried out in RStudio Version 1.3.1093 (RStudio, Inc., Boston, MA, USA). Data were mean centering or log transformation to satisfy normality and homoscedasticity as necessary.

## 5. Conclusions

Our study concluded that the underground part, including the root system and soil ecosystem, played an important role in K mitigation on drought stress. The stressed rapeseed with suitable K supply not only showed an improved potential of elongating root length, boosting root density, and balancing root-shoot ratio to increase water uptake, but also stimulated organic acids secretion to enhance nutrient acquisition and utilization. However, this alleviation had a hormesis effect, which means that K has a continuous promotion in the appropriate range but severely inhibits plant growth once the application is excessive. Furthermore, the positive effects of K were more effective in water-limited conditions. K application contributed to microbial community development by enriching nutrients and neutralizing pH to establish a healthy soil environment, which could help plants maintain resistance and tolerance against drought. Our study quantified the potential ability of organic acids on AK activation to withstand water deficit, but fully understand the underlying mechanism of root exudates responses of climate changes requires deep interpretation on the molecular level.

## Data Availability

The data presented in this study is contained within the article.

## Supplementary Materials

The following are available online at https://www.mdpi.com/2218-1989/11/3/131/s1, Figure S1: Root bags in seeds spotting and soil sampling period, Figure S2: Root bags in seeds spotting and soil sampling period.
