# Supplementary material for: Potassium Improves Drought Stress Tolerance in Plants by Affecting Root Morphology, Root Exudates, and Microbial Diversity
Source: Metabolites. 2021 Feb 24;11(3):131. doi: 10.3390/metabo11030131 (PMC7996290; doi:10.3390/metabo11030131)
Supplement: Supplementary file 1 [file metabolites-11-00131-s001.pdf]

Experimental photos with roots bags

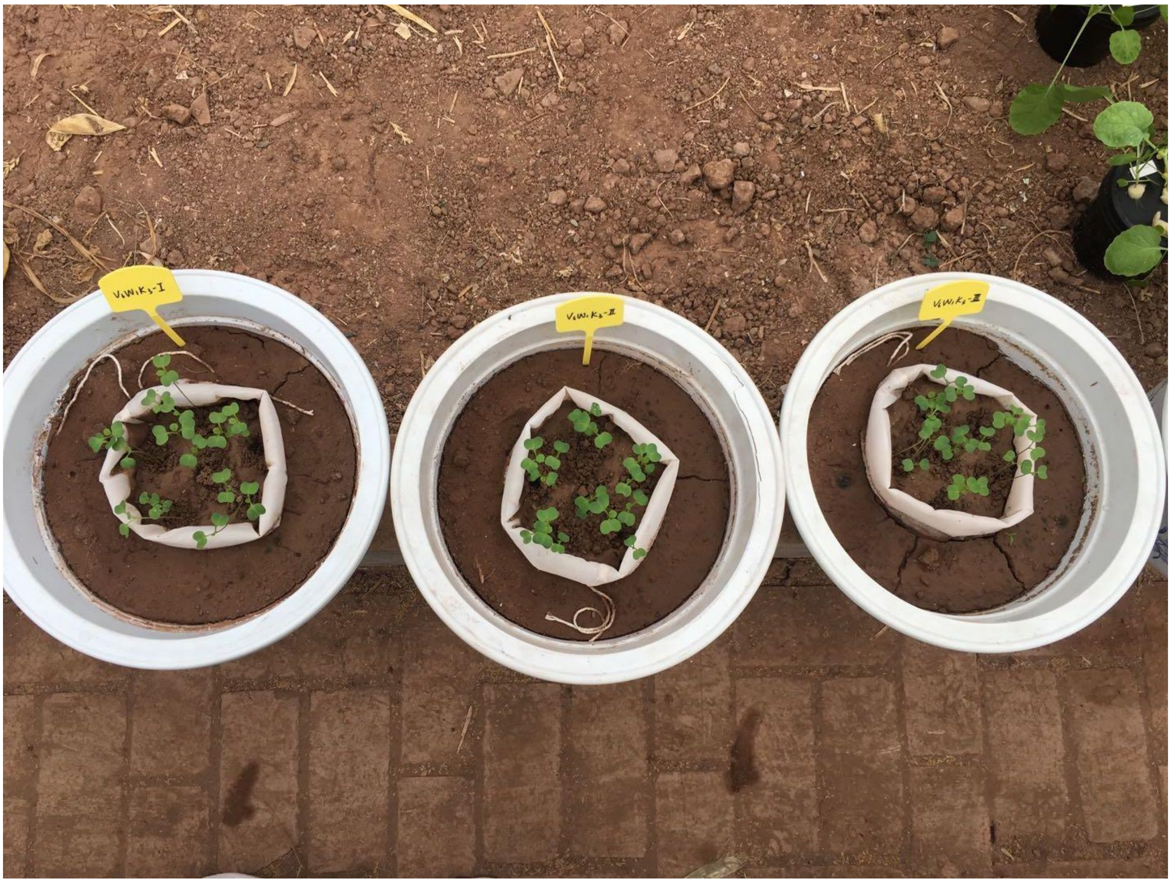

Figure S1: Root bags in seeds spotting and soil sampling period.

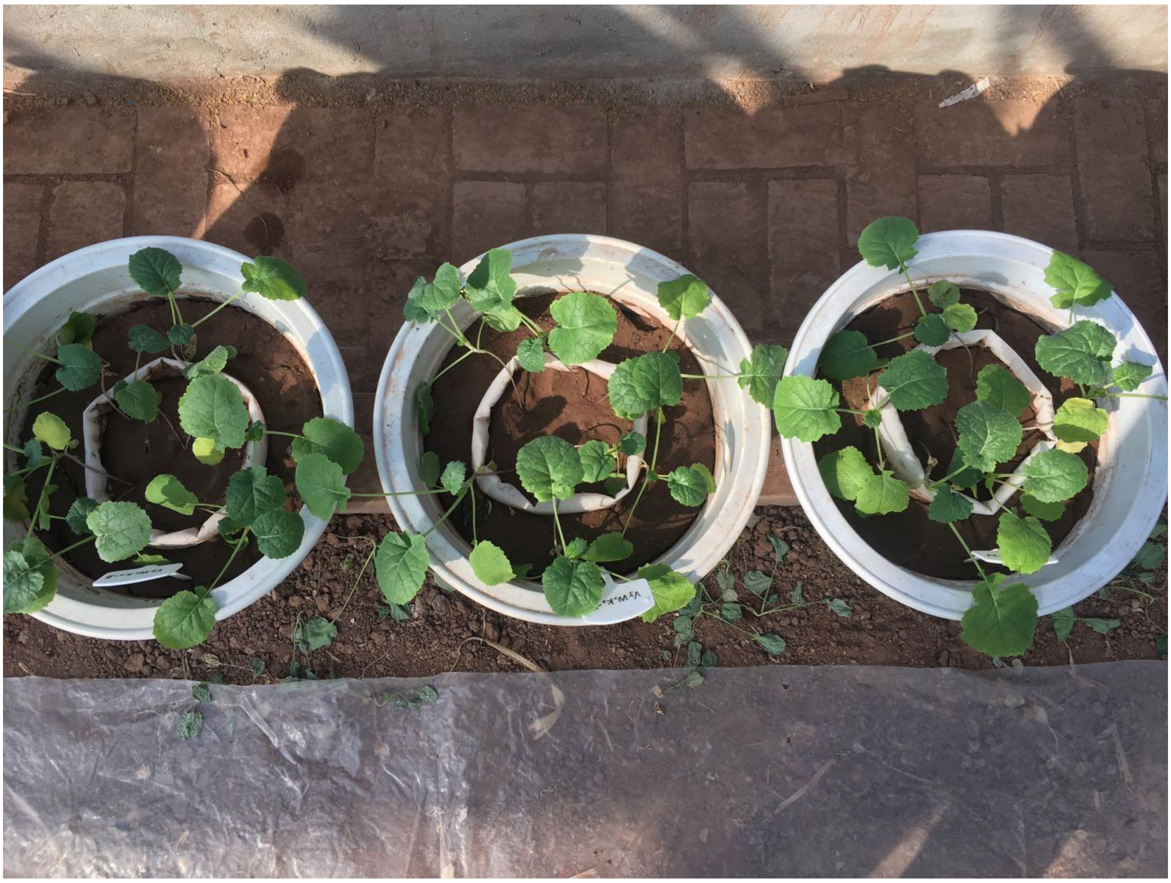

Figure S2: Root bags in seeds spotting and soil sampling period**.**
